# Knee landmarks detection via deep learning for automatic imaging evaluation of trochlear dysplasia and patellar height

**DOI:** 10.1007/s00330-024-10596-9

**Published:** 2024-02-10

**Authors:** Roberto M. Barbosa, Luís Serrador, Manuel Vieira da Silva, Carlos Sampaio Macedo, Cristina P. Santos

**Affiliations:** 1 Center of MicroElectroMechanical Systems (CMEMS), University of Minho, Guimarães, Portugal; 2 MIT Portugal Program, School of Engineering, University of Minho, Guimarães, Portugal; 3 Department of Orthopaedics, Trofa Saúde Braga Centro Hospital, Braga, Portugal; 4 Department of Radiology, Trofa Saúde Braga Centro Hospital, Braga, Portugal; 5 LABBELS - Associate Laboratory, Braga/Guimarães, Portugal

**Keywords:** Knee, Patellofemoral joint, Patellar dislocation, Magnetic resonance imaging, Deep learning

## Abstract

**Objectives:**

To develop and validate a deep learning–based approach to automatically measure the patellofemoral instability (PFI) indices related to patellar height and trochlear dysplasia in knee magnetic resonance imaging (MRI) scans.

**Methods:**

A total of 763 knee MRI slices from 95 patients were included in the study, and 3393 anatomical landmarks were annotated for measuring sulcus angle (SA), trochlear facet asymmetry (TFA), trochlear groove depth (TGD) and lateral trochlear inclination (LTI) to assess trochlear dysplasia, and Insall-Salvati index (ISI), modified Insall-Salvati index (MISI), Caton Deschamps index (CDI) and patellotrochlear index (PTI) to assess patellar height. A U-Net based network was implemented to predict the landmarks’ locations. The successful detection rate (SDR) and the mean absolute error (MAE) evaluation metrics were used to evaluate the performance of the network. The intraclass correlation coefficient (ICC) was also used to evaluate the reliability of the proposed framework to measure the mentioned PFI indices.

**Results:**

The developed models achieved good accuracy in predicting the landmarks’ locations, with a maximum value for the MAE of 1.38 ± 0.76 mm. The results show that LTI, TGD, ISI, CDI and PTI can be measured with excellent reliability (ICC > 0.9), and SA, TFA and MISI can be measured with good reliability (ICC > 0.75), with the proposed framework.

**Conclusions:**

This study proposes a reliable approach with promising applicability for automatic patellar height and trochlear dysplasia assessment, assisting the radiologists in their clinical practice.

**Clinical relevance statement:**

The objective knee landmarks detection on MRI images provided by artificial intelligence may improve the reproducibility and reliability of the imaging evaluation of trochlear anatomy and patellar height, assisting radiologists in their clinical practice in the patellofemoral instability assessment.

**Key Points:**

*• Imaging evaluation of patellofemoral instability is subjective and vulnerable to substantial intra and interobserver variability.*

• *Patellar height and trochlear dysplasia are reliably assessed in MRI by means of artificial intelligence (AI).*

• *The developed AI framework provides an objective evaluation of patellar height and trochlear dysplasia enhancing the clinical practice of the radiologists.*

## Introduction

The multifactorial origin of anterior knee pain (AKP) leads to a demanding diagnostic imaging of the patellofemoral joint (PFJ). AKP can be caused by PFJ misalignment and often by muscle weakness, leading to patellofemoral instability (PFI) [1]. The main risk factors of PFI are trochlear dysplasia, excessive patellar height and excessive lateralisation of the anterior tibial tuberosity [2]. Trochlear dysplasia is highlighted by Dejour et al as one of the most predisposing factors for this disease [3]. A percentage of 85 to 96% of patients with recurrent PFI presents a dysplastic trochlea [4]. Patella *alta*, designation for an excessive patellar height, is observed in about 25% of the patients with acute patellar dislocation [5].

Several indices have been addressed in the literature to assess the main risk factors of PFI. Regarding trochlear dysplasia, the main PFI indices described in the literature using cross-sectional studies are sulcus angle (SA) [6], trochlear facet asymmetry (TFA) [7], trochlear groove depth (TGD) [8] and lateral trochlear inclination (LTI) [9]. Insall-Salvati index (ISI) [10], modified Insall-Salvati index (MISI) [11], Caton Deschamps index (CDI) [12] and patellotrochlear index (PTI) [13] have been addressed for patellar height assessment.

PFI index measurements are prone to high intra- and interobserver variability originated by the subjective anatomical landmarks identification, and it is described by the radiologists as a tedious and time-consuming task [14, 15]. Some traditional mathematical models have been addressed in the literature to eliminate the variability originated by manual annotations for measuring ISI [16], tibial tubercle and patellar lateralisation [17] and patellar tilt and lateralisation [18]. The introduction of deep learning (DL) has revolutionised the medical imaging analysis field aiming for objective and automatic detection of anatomical landmarks. Two works were found in the literature applying DL-based approaches to directly predict the landmarks’ locations and, consequently, automate PFI index measurements. Ye et al proposed a framework to automatically assess patellar height using radiographic images [19], and Tuya et al presented a study addressing the automatic measurement of SA and patellar tilt and lateralisation in plain radiographs [20]. However, radiographic images are limited to assess the PFJ. Several authors have been committed to uniform the PFI assessment with magnetic resonance imaging (MRI), demonstrating that it will be the future standard [12, 21–24].

This manuscript aims to develop and evaluate an automatic tool resorting to DL-based algorithms for assessing trochlear dysplasia and patellar height. It is proposed to automatically detect the landmarks’ locations and compute the index measurements in knee MRI slices. This study provides research evidence concerning the promising use of DL-based strategies to assist in the measurements of PFI indices, supporting the radiologist in such demanding and time-consuming task and improving the reliability of the process with more objective measurements.

## Materials and methods

### Dataset

The study was approved by the Ethics Committee of Trofa Saúde, and the requirement for informed consent was waived. The dataset used in this retrospective study comprises axial and sagittal knee MRI scans. All images used in the study were acquired at 7 different clinical centres of the Trofa Saúde health group, from April 2018 to June 2021. All data were anonymised for analysis.

Inclusion criteria included patients whose images were acquired following the protocol implemented at the institution for assessing the patellofemoral joint that comprises diversified MRI sequences, including proton density (PD), and T1- and T2-weighted images in the axial, sagittal and coronal planes. Healthy and pathological knees were included in the dataset. Pathological knees comprised trochlear dysplasia, patella *alta* and excessive lateralisation of tibial tubercle. Exclusion criteria covered all knees that had undergone medial patellofemoral ligament reconstruction or tibial tubercle transfer due to screw artifacts.

**Fig. 1:**
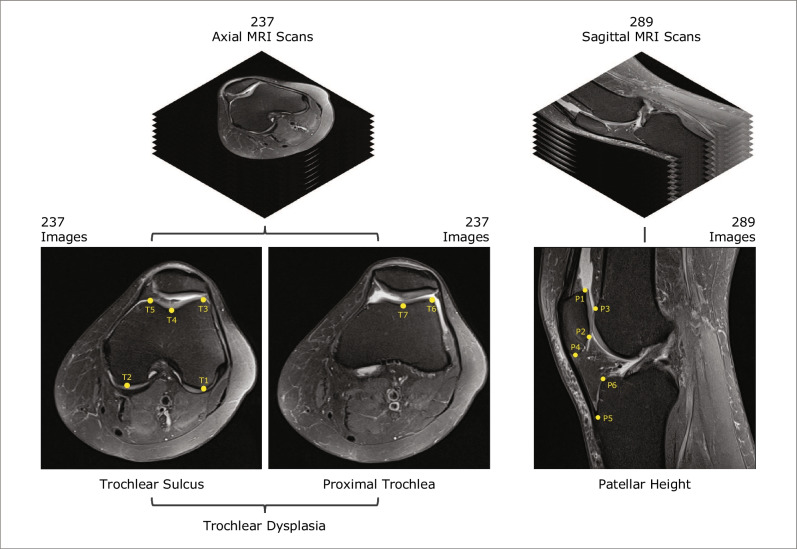
Representation of the dataset used in the study. All landmarks were named as shown. Landmarks annotations in “Trochlear Sulcus” image: T1, the posterior aspect of the lateral femoral condyle; T2, the posterior aspect of the medial femoral condyle; T3, the anterior aspect of the lateral trochlear facet; T4, the deepest point of the trochlear sulcus; T5, the anterior aspect of the medial trochlear facet. Landmarks annotations in “Proximal Trochlea” image: T6, the lateral margin of the lateral trochlear facet; T7, the medial margin of the lateral trochlear facet. Landmarks annotations in “Patellar Height” image: P1, the most proximal aspect of the patella; P2, the most distal aspect of the patellar articular surface; P3, the most proximal margin of the trochlear cartilage; P4, the patella apex; P5, the insertion of the patellar tendon on the anterior tibial tuberosity; P6, the anterior aspect of the tibia plateau

A total of 140 knees (including 28 with trochlear dysplasia, 34 with patella *alta* and 23 with excessive lateralisation of tibial tubercle) from 95 patients were reviewed, comprising 68 left knees and 72 right knees. Multiple PD and T1- and T2-weighted images of both axial and sagittal sequences were included in the dataset, resulting in 237 axial (119 left; 118 right–153 PD; 84 T1) and 289 sagittal (139 left; 150 right–144 PD; 137 T1; 8 T2) MRI sequences. Taking this dataset, an expert musculoskeletal radiologist selected two images from each axial sequence: the image 3 cm above the femorotibial joint line which contains the intercondylar groove with an appearance of a Roman arch, where the cartilaginous trochlear surface is completely exposed (“Trochlear Sulcus”, Fig. 1), and the first craniocaudal image showing trochlear cartilage (“Proximal Trochlea”, Fig. 1). In the sagittal sequences, the image containing the longest axis of the patella was selected from each sequence (“Patellar Height”, Fig. 1). The final dataset contains 763 images (237 “Trochlear Sulcus”; 237 “Proximal Trochlea”; 289 “Patellar Height”). After selecting the images, the same radiologist performed the labelling. A total of 3393 landmarks were annotated. The dataset representation and the landmarks are presented in Fig. 1.

### Data preparation

The images from 13 patients of the “Trochlear Sulcus” and “Proximal Trochlea” subsets were selected to the hold-out test sets, resulting in 37 images (19 left; 18 right) for each one, ensuring the proportions of left and right knees. The remaining data were randomly split into training set (*n* = 160, 80%), and validation set (*n* = 40, 20%). Concerning the “Patellar Height” subset, a hold-out test set was created including the sagittal images from the same 13 patients, resulting in 39 images (19 left; 20 right), also ensuring the proportions of left and right knees. The same ratio was followed to randomly split the remaining data into training set (*n* = 200, 80%), and validation set (*n* = 50, 20%).

All images were resized to 320 × 320 pixels and normalised to [0,1] before training. In order to improve the detection performance and to reduce the overfitting, data augmentation method during training was implemented, by applying random pixel multiplication in the range [1.25, 0.75], random pixel shift of [− 0.15, 0.15], random flip on the left–right axis, random zoom of [0.9, 1.1], random rotation of [− 10, 10]°, random translation in vertical and horizontal axes of [− 5, 5]% and Gaussian noise and Gaussian blur.

### Automatic PFI index measurements

**Fig. 2:**
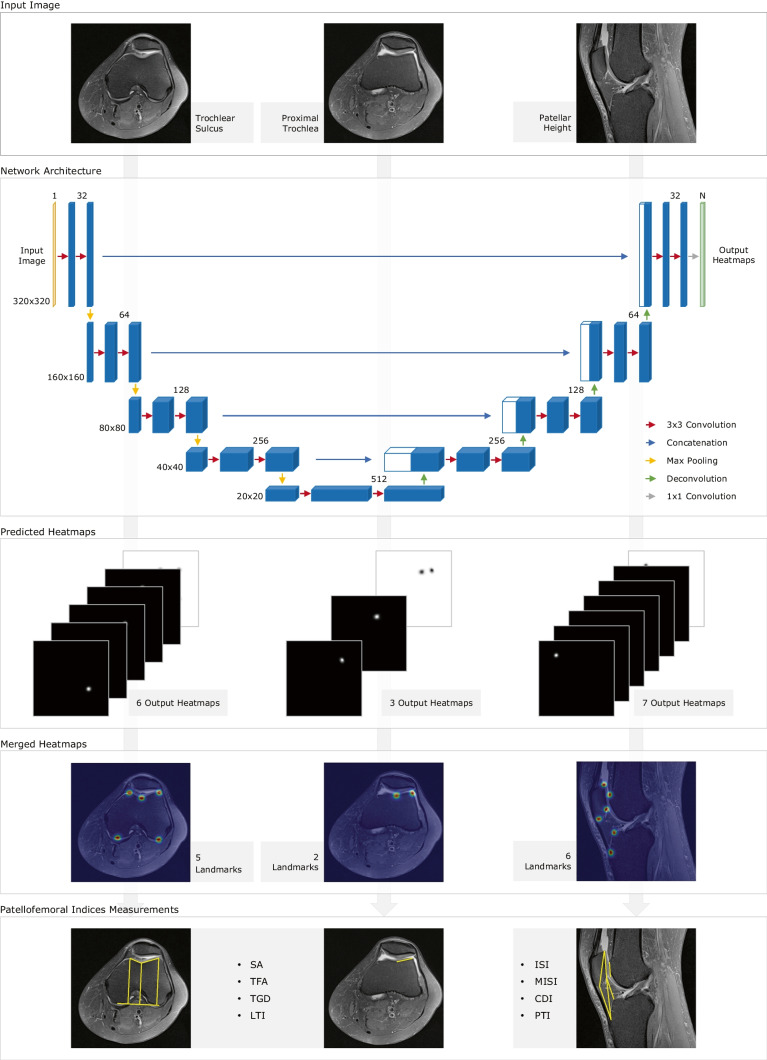
Flowchart of the study design

**Fig. 3:**
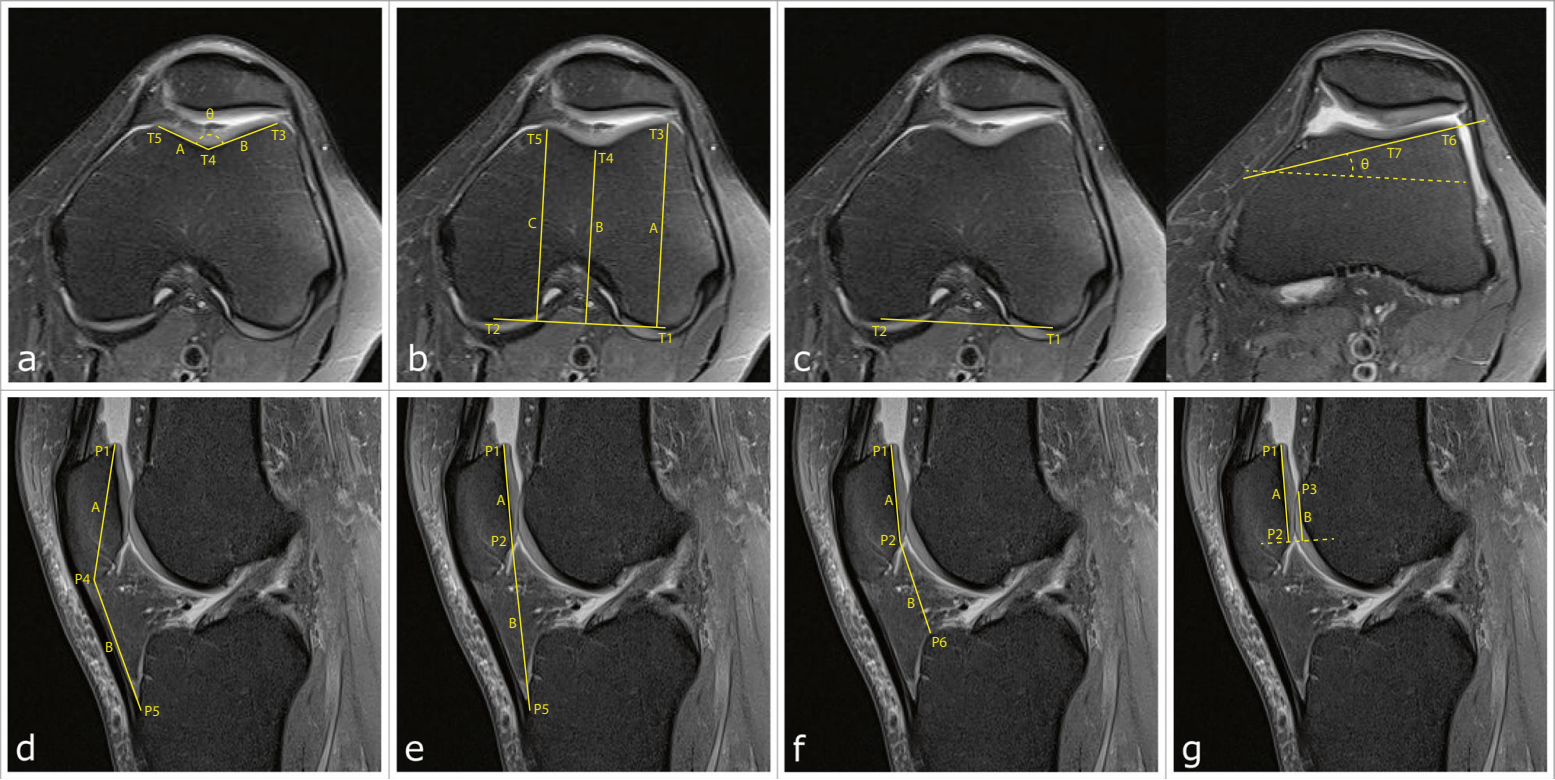
Axial and sagittal fat-saturated proton density weighted turbo spin-echo magnetic resonance images demonstrating the measurements of the indices to assess trochlear dysplasia and patellar height, respectively. The axial slice 3 cm above the femorotibial joint line that includes the intercondylar groove with an appearance of a Roman arch was selected to measure sulcus angle (θ) and trochlear facet asymmetry (A/B) (**a**), and trochlear groove depth ((A + C)/2-B) (**b**). The first craniocaudal image showing trochlear cartilage was also selected for measuring lateral trochlear inclination (θ) (**c**). The sagittal slice showing the longest axis of the patella was selected to measure Insall-Salvati index (B/A) (**d**), modified Insall-Salvati index (B/A) (**e**), Caton Deschamps index (B/A) (**f**) and patellotrochlear index (B/A) (**g**)

Figure 2 shows the flowchart of the study. Three models were generated to detect the landmarks on (1) “Trochlear Sulcus”, (2) “Proximal Trochlea” and (3) “Patellar Height”. These models were trained using the same U-Net based network architecture, presented in Fig. 2. Mathematical relationships are then computed using the predicted landmarks’ coordinates to estimate the PFI indices values. Figure 3 demonstrates the methodology to measure the chosen PFI indices for assessing trochlear dysplasia and patellar height.

#### Labelling processing

This manuscript addresses the automatic landmarks’ coordinate detection problem. The discrete coordinates of each landmark were modelled to a heatmap. Compared to direct landmarks’ coordinate regression approaches, heatmap regression methods have demonstrated to be more suitable for this purpose, converting it into a pixel-to-pixel classification problem. It provides the probability of each pixel containing the specific landmark, emphasising the importance of the landmark spatial location, helping the network to be more aware of the relative positions among the landmarks [25–30].

One heatmap channel of 320 × 320 pixels was generated for each landmark, with a Gaussian distribution centred in the landmark location [26, 28]. The heatmap values were normalised to [0,1] so that the maximum of the Gaussian distribution is 1, which corresponds to the exact location of the corresponding landmark. Therefore, the heatmap has a value of 0 in the background, and it increases gradually at the surrounding of the landmark location. This results in a class imbalance problem since the background area is much higher than the landmark Gaussian distribution. To overcome this, a shared heatmap background channel was generated, ensuring that the sum of all heatmaps is 1 in each pixel. Each heatmap channel is represented by *H*. Being *L* the number of landmarks to detect, the number of heatmap channels is *L* + 1, as follows:

1 $${H}_{i} \left(x,y\right)=\left\{\begin{array}{c}{e}^{\left(- \frac{1}{2{\sigma }^{2}} \left({\left(x - a\right)}^{2} +{ \left(y - b\right)}^{2}\right)\right)} , i=1,\dots ,L\\ 1-\sum\limits_{j=1}^{L}{H}_{j }\left(x,y\right) , i=L+1\end{array}\right.$$

where (*a*, *b*) is the centre of the Gaussian distribution, and the last heatmap channel (${H}_{L+1}$), corresponds to the shared background by all heatmaps with the landmark distributions [26, 29, 31].

#### Network architecture

Three U-Net based models were trained for “Trochlear Sulcus”, “Proximal Trochlea” and “Patella Height”. Each one contains different input images and a different number of output channels, 6, 3 and 7, respectively, which correspond to the number of landmarks to detect plus the background channel. Each model is composed by a contracting and expanding path, like the original U-Net architecture (Fig. 2) [32]. Its symmetrical shape enables to capture the context information in the embedded encoder, and the embedded decoder allows precise location [32]. The encoder consists of the repetitive application of two convolutions, with a kernel size of 3 × 3 and a He normal distribution for the kernel initialisation, followed by a rectified linear unit (ReLU) and a 2 × 2 max pooling layer in each level. A dropout layer was included after the first convolution in each level. Dropout layers of 0.1, 0.1, 0.2, 0.2 and 0.3 were applied at each level of the contracting path, respectively. Dropout layers have a significative role in the learning process of the model, capturing more robust features [33]. The number of feature channels doubles at each level, starting at 32.

Each step in the decoder consists of a 2 × 2 transposed convolution (also known as deconvolution) layer, halving the number of features channels, followed by a concatenation of the feature map, of the corresponding level, from the contracting path. Two 3 × 3 convolution layers were then applied, with the same kernel size and initialisation, and the same activation ReLU. Dropout layers were also added in each level, after the first convolution. Decreasing dropouts of 0.2, 0.2, 0.1 and 0.1 were applied at each level. The last layer consists of a 1 × 1 convolution with softmax activation, whose output corresponds to a multi-channel heatmap, one heatmap for each landmark and one for the background [32].

### Training

The categorical cross entropy function was used to train the model, by penalising the deviation of each pixel prediction from 1, by the logarithmic function:

2 $$Loss = -\sum\limits_{i=1}^{\begin{array}{c}output\\ size\end{array}}{t}_{i}\times {\text{log}}{p}_{i}$$

where ${p}_{i}$ is the *i*th scalar value in the model prediction, and ${t}_{i}$ is the corresponding target value, the ground truth. Error decreases logarithmically as ${p}_{i}$ tends to ${t}_{i}$ [32].

Google Colaboratory platform was used to train the network with a batch size of 16 [34]. The network was trained with Adam optimiser with a fixed learning rate of 0.001 during 200 epochs.

#### PFI index measurements

Given the output of the models, the coordinates of the landmarks were extracted from the corresponding output heatmaps by finding its maximum pixel value. After obtaining the landmarks’ coordinates, automatic calculations of all PFI indices addressed were made according to the description of Fig. 3.

### Evaluation metrics

All data were analysed using Microsoft Excel (version 16.61) and IBM SPSS Statistics (version 28). The data were tested for normality, with statistical significance defined at *p* < 0.05.

In this manuscript, evaluation metrics were used: (1) to evaluate the performance of the developed models to detect the landmarks, using the successful detection rate (SDR), and the mean absolute error (MAE) with the standard deviation (STD) metrics, and (2) to evaluate the reliability of the proposed method to automatically estimate the PFI indices values, using the intraclass correlation coefficient (ICC), and the MAE with the STD. The outcomes of the trained network models, using the hold-out test sets as input, were analysed. The landmarks’ coordinates were obtained by extracting the location of the maximum pixel value for each corresponding output heatmap.

SDR corresponds to the percentage of successfully detected landmarks within a specific precision range from the ground truth location [26–28, 35, 36]. Percentage of correct keypoints (PCK) is used in some related works, which is equivalent to SDR [19, 20, 37]. The accuracy of each landmark detection was evaluated for each range *R* of 1 mm, 1.5 mm, 2 mm, 2.5 mm, 3 mm, 4 mm and 5 mm, as follows:

3 $${SDR}_{R}=\frac{\# \left\{i:\Vert {t}_{i}-{p}_{i}\Vert <R\right\}}{\# N}$$

where $\Vert {t}_{i}-{p}_{i}\Vert$ designates the Euclidean distance between ground truth and the model prediction, *N* corresponds to the test set samples and *i* ∈ *N*.

MAE and its STD were calculated to evaluate both the performance of the developed models to detect each landmark, and to evaluate the reliability of the proposed framework to automatically measure the addressed PFI indices. MAE is obtained by:

4 $$MAE = \frac{1}{N} \sum\limits_{i=1}^{N} \Vert {t}_{i}-{p}_{i}\Vert$$

ICC was also employed as an evaluation metric of the reliability of the proposed framework to automatically estimate the PFI indices. It reflects both degree of correlation and agreement between measurements [38]. The grades of reliability, according to ICC, are classified into poor (values less than 0.5), moderate (values between 0.5 and 0.75), good (values between 0.75 and 0.9) and excellent (values greater than 0.9) [38].

## Results

### Demographic information

Concerning the “Trochlear Sulcus” and “Proximal Trochlea” subsets, the mean age was 25.68 ± 10.89 years (range, 6 to 59 years) for training and validations data sets, and 26.27 ± 11.40 years (range, 12 to 48 years) for the test data set. All data sets contain a higher percentage of females, ranging from 68% in the training and validation data sets to 54% in the test data set. Regarding the “Patellar Height” subset, the mean age was 26.24 ± 11.51 years (range, 6 to 59 years) for training and validations data sets, and 26.31 ± 11.40 years (range, 12 to 48 years) for the test data set. The percentage of females ranges from 69% in the training and validation data sets to 59% in the test data set. All data sets had a balanced laterality distribution. The data distribution is presented in Table 1.

**Table 1:** Data distribution of training, validation and test data sets

	“Trochlear Sulcus” and “Proximal Trochlea”		“Patellar Height”	
Data sets	Training and validation	Test	Training and validation	Test
No. of images	200	37	250	39
Age (mean ± STD)	25.68 ± 10.89	26.27 ± 11.40	26.24 ± 11.51	26.31 ± 11.40
Sex				
Male (%)	64 (32)	17 (46)	77 (31)	16 (41)
Female (%)	136 (68)	20 (54)	173 (69)	23 (59)
Side				
Left (%)	100 (50)	19 (51)	120 (48)	19 (49)
Right (%)	100 (50)	18 (49)	130 (52)	20 (51)

### Performance of landmarks detection

**Fig. 4:**
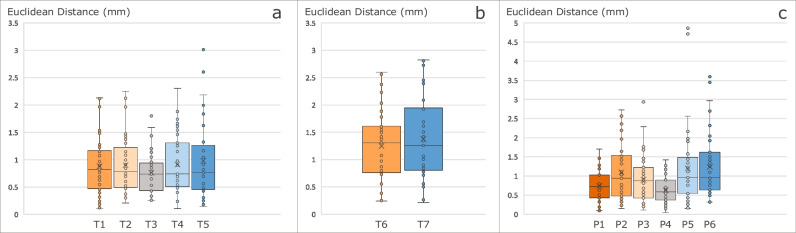
Box plots of Euclidean distances between ground truth and predicted landmarks. Landmarks on “Trochlear Sulcus” (**a**). Landmarks on “Proximal Trochlea” (**b**). Landmarks on “Patellar Height” (**c**)

Regarding the performance of the trained models to predict the landmarks’ locations, Fig. 4 presents the box plots of the localisation error that correspond to the Euclidean distances between ground truth and the predictions for each landmark, “Trochlear Sulcus” (T#1–5), “Proximal Trochlea” (T#6–7) and “Patellar Height” (P#1–6).

**Fig. 5:**
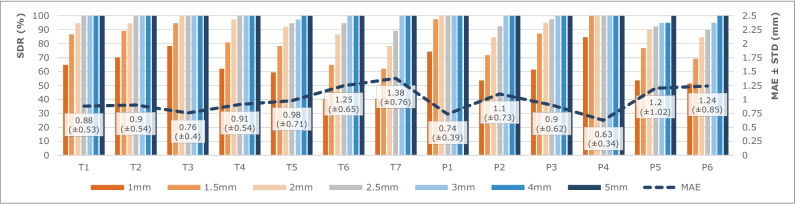
Graphic representation of successful detection rate (SDR) and mean absolute error (MAE) of all patellofemoral landmarks

SDR and MAE of the 13 landmarks are shown in Fig. 5. The minimum MAE was obtained for landmarks P4, with a value of 0.63 ± 0.34 mm. On the other hand, the landmarks T7 have presented a MAE of 1.38 ± 0.76 mm, which corresponds to the maximum value of MAE among all predicted landmarks.

Looking at SDR results, there are 9 landmarks with SDR higher than 75%, presenting a Euclidean distance inferior to 1.5 mm from the ground truth locations. Considering the 2.5 mm range, there are 11 landmarks with SDR higher than 90%. All landmarks were detected within the range of 5 mm. The P4 landmarks were all successfully detected within the 1.5-mm range.

**Fig. 6:**
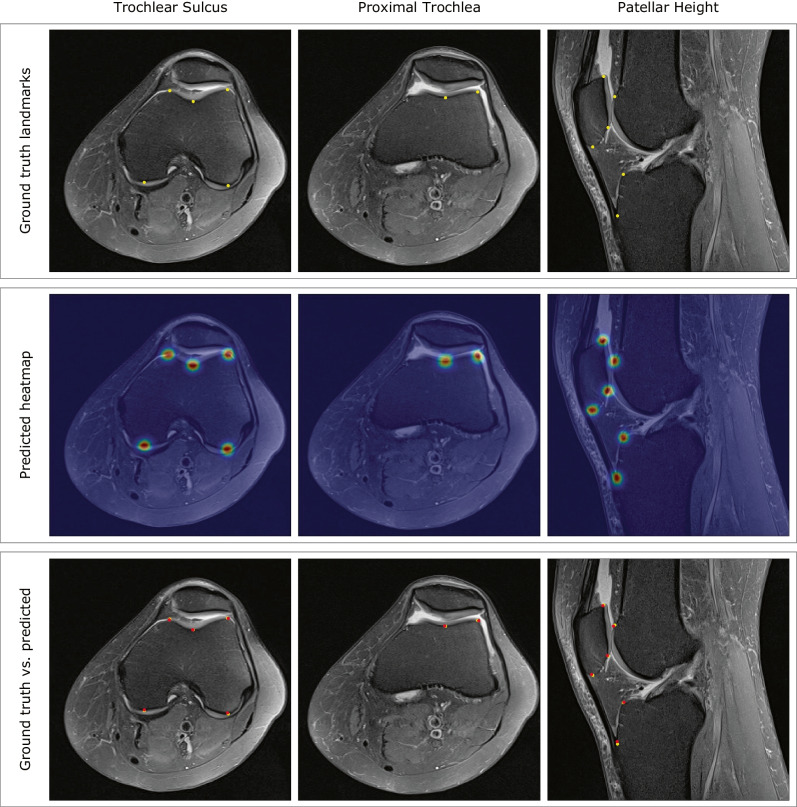
Representative examples illustrating the landmarks detection results achieved by each developed model. First row contains the ground truth landmarks, identified by the yellow colour. The predicted landmark heatmaps are shown in second row. Third row presents the superposition of the ground truth and the predicted landmarks’ locations, identified by the red colour

A representative example of the landmarks detection results achieved by each developed model is presented in Fig. 6.

### Reliability of automatic PFI index measurements

The ICC between the PFI indices values obtained from the ground truth landmarks and from the predicted landmarks was calculated to conclude about the reliability of the proposed framework to automatically measure the addressed PFI indices. Considering the results presented in Table 2, an excellent reliability was observed for the measurements of LTI (ICC = 0.907), TGD (ICC = 0.918), ISI (ICC = 0.985), CDI (ICC = 0.921) and PTI (ICC = 0.967). All remaining PFI index measurements have achieved a good reliability, with an ICC value higher than 0.75.

**Table 2:** Intraclass correlation coefficient (ICC), 95% confidence intervals (CI) and mean absolute error (MAE) of proposed framework for automatic measurements of patellofemoral instability indices

Patellofemoral indices	ICC	95% CI	MAE ± STD
Trochlear dysplasia			
LTI	0.907	(0.814; 0.953)	2.078 ± 1.601º
SA	0.878	(0.776; 0.935)	3.535 ± 2.944º
TFA	0.781	(0.608; 0.882)	0.059 ± 0.041
TGD	0.918	(0.847; 0.957)	0.500 ± 0.453 mm
Patellar height			
ISI	0.985	(0.971; 0.992)	0.035 ± 0.027
MISI	0.868	(0.762; 0.929)	0.087 ± 0.084
CDI	0.921	(0.854; 0.958)	0.064 ± 0.056
PTI	0.967	(0.925; 0.984)	0.032 ± 0.027

## Discussion

This work proposes an automatic DL-based framework for trochlear dysplasia and patellar height assessment. The implemented solution resorts to a U-Net based network architecture to automatically detect the anatomical landmarks in axial and sagittal MRI slices of the PFJ, following the state-of-the-art indications [19, 26, 27, 29–32]. The results have shown that the proposed method is reliable to automatically measure the main PFI indices addressed in the literature, when compared to the measurements values provided by an expert musculoskeletal radiologist, and it represents a substantial contribution in assisting radiologists by saving time and reducing task complexity.

In order to evaluate the performance of the models to predict the landmarks’ locations, SDR and MAE metrics were used, in accordance with the related works found in the literature [19, 20, 26, 27, 30, 36]. The obtained results are summed up in Fig. 5. Overall, the achieved values are adequate, with the highest MAE of 1.38 ± 0.76 mm for the landmark T7. Chen et al have reported that a mean distance up to 2.98 mm for landmark location is satisfactory for clinical analyses [16]. In the range of 3 mm, all landmarks obtained a SDR superior to 97.3% in “Trochlear Sulcus”, all “Patellar Height” landmarks obtained at least 94.87% and all landmarks were successfully detected in the “Proximal Trochlea”. According to the grades of reliability defined in [38], it is reasonable to conclude that the LTI, TGD, ISI, CDI and PTI can be measured with excellent reliability (ICC > 0.9), and SA, TFA and MISI can be measured with good reliability (ICC > 0.75), by the proposed framework for automatic PFI index measurements. Several studies have been conducted to investigate the intra and interobserver reliability of PFI index measurement. The proposed framework has proved that its objective measurements improve the significative variability registered in the literature for manual measurements of PFI indices [2, 14, 23, 24, 39–44]. The achieved reliability is better than the overall values presented in the literature for manual measurements.

Studies in the literature addressing the use of DL-based models to automate landmark detection for PFI index measurements are scarce. Nevertheless, two related works are found using radiographic images [19, 20]. Ye et al proposed a method to automatically measure four patellar height indices, including ISI and CDI [19], and E et al addressed the automatic measurement of SA and three PFI indices related to patellar tilt and lateralisation [20]. Compared to these state-of-the-art studies, the proposed framework offers a more comprehensive evaluation of the PFJ. It includes the assessment of both patellar height and trochlear dysplasia using the widely accepted medical imaging technique, MRI. Only SA in the work conducted by E et al obtained a higher ICC value when compared to our framework [20]. The use of the radiographic Laurin view in the mentioned study provides a closer view of the trochlea, and these discrepancies in imaging modalities may have contributed to the observed outcomes. Additionally, the inclusion of patients with severe dysplastic trochleae in our dataset may explain the obtained results. Further research that incorporates a larger number of images depicting dysplastic trochleae will allow the network to better learn the key features present in these images, thereby enhancing the outcome of the proposed framework.

The presented work has some limitations that should be considered in further studies. One concern is related to the small dataset used to conduct this work. Despite the good results achieved in this work, a larger and more varied dataset would enable more robust models. Other issue is related to the positioning of all landmarks in the same slice to assess the patellar height. Since the dataset contain pathological patients, the sagittal image of those who present an excessive lateralisation of the patella does not contain the perfect location of the anterior tibial tuberosity, the most anterior aspect of the tibial plate and the major axis of the patella. A trade-off between these morphological aspects was made to select the most suitable slice to consider in the dataset. Further studies must include the exploration of automatic 3D approaches for landmarks detection.

## Conclusions

This work presents an approach using DL-based models to automatically assess trochlear dysplasia and patellar height using MRI slices. The developed framework allows to automatically detect the patellofemoral landmarks and to estimate the values of LTI, SA, TFA, TGD, ISI, MISI, CDI and PTI. All PFI indices achieved an ICC value higher than 0.75, corroborating that it is proposed a reliable system to automatically measure the PFI indices resorting to MRI with promising applicability in the clinical practice of the radiologists. It consists of a significant contribution to assist the tedious and demanding imaging evaluation of PFI and tackling the reported variability of the procedure.

## Abbreviations

AKP: Anterior knee pain
CDI: Caton-Deschamps index
CT: Computed tomography
DL: Deep learning
ICC: Intraclass correlation coefficient
ISI: Insall-Salvati index
LTI: Lateral trochlear inclination
MAE: Mean absolute error
MISI: Modified Insall-Salvati index
MRI: Magnetic resonance imaging
PCK: Percentage of correct keypoints
PD: Proton density
PFI: Patellofemoral instability
PFJ: Patellofemoral joint
PTI: Patellotrochlear index
ReLU: Rectified linear unit
SA: Sulcus angle
SDR: Successful detection rate
STD: Standard deviation
TFA: Trochlear facet asymmetry
TGD: Trochlear groove depth

## Declarations

### Guarantor

The scientific guarantor of this publication is Professor Cristina P. Santos.

### Statistics and biometry

No complex statistical methods were necessary for this paper.

### Methodology

• retrospective

• diagnostic study

• multicentre study
